# Versatility of High-Velocity Nasal Insufflation (HVNI) in respiratory weaning: A case series demonstrating three distinct clinical objectives

**DOI:** 10.1016/j.rmcr.2026.102492

**Published:** 2026-08-27

**Authors:** Iacopo Cappellini, Vittorio Pavoni

**Affiliations:** Department of Emergency and Critical Care Medicine, Section of Anesthesiology and Intensive Care, Ospedale Santo Stefano, Prato, Italy

**Keywords:** High-velocity nasal insufflation, High-flow nasal therapy, Weaning, Extubation, COPD, Hypercapnic respiratory failure, Tracheostomy, Noninvasive ventilation, Airway clearance, Intensive care

## Abstract

High-Velocity Nasal Insufflation (HVNI) is an advanced form of high-flow nasal therapy that delivers heated, humidified oxygen at high velocities through a small-bore nasal cannula, generating efficient extrathoracic dead space washout, mild positive end-expiratory pressure (PEEP), and enhanced patient comfort. While Non-Invasive Positive Pressure Ventilation (NiPPV) remains the reference treatment for acute hypercapnic respiratory failure, its use is frequently limited by patient intolerance. This case series presents three critically ill patients in whom HVNI was deployed during the respiratory weaning phase to achieve three distinct clinical objectives: (1) prophylactic stabilization to prevent post-extubation failure in a high-risk COPD patient; (2) rescue therapy for NiPPV intolerance with simultaneous airway clearance; and (3) bridging functional recovery during prolonged tracheostomy weaning. All patients achieved their targeted clinical endpoints without adverse events. These cases illustrate the clinical versatility of HVNI as an adaptable tool in the intensivist's armamentarium for individualized respiratory weaning strategies.

## Highlights

• HVNI was used during respiratory weaning to meet three distinct clinical objectives.
• HVNI provided prophylactic post-extubation stabilization in a high-risk COPD patient.
• HVNI served as rescue therapy for NiPPV intolerance while allowing airway clearance.
• HVNI enabled mobilization and functional recovery during prolonged tracheostomy weaning.

## 1 Introduction

High-Velocity Nasal Insufflation (HVNI) is a refine form of high-flow nasal cannula (HFNC) therapy that delivers heated (34–37°C), humidified gas at flow rates up to 45 L/min through a smaller-bore nasal cannula [1]. The smaller cannula bore generates higher kinetic energy at equivalent flow rates, rapidly flushing the extrathoracic dead space of carbon dioxide, reducing the work of breathing, and providing a mild, flow-dependent positive end-expiratory pressure (PEEP) [2,3]. These physiological effects—dead space washout, accurate FiO_2_ delivery, low-level PEEP generation, and enhanced mucociliary clearance through optimal humidification—are well-established mechanisms of high-flow nasal therapies [[4], [5], [6]].

Non-Invasive Positive Pressure Ventilation (NiPPV) remains the cornerstone of treatment for acute hypercapnic respiratory failure, particularly in COPD exacerbations [7]. However, its clinical application is frequently limited by patient intolerance due to mask discomfort, claustrophobia, and interference with secretion clearance and oral intake [8]. Recent randomized clinical trials have demonstrated that HVNI is non-inferior to NiPPV in the treatment of undifferentiated respiratory failure and moderate acute exacerbations of COPD with hypercapnic respiratory failure, with significantly improved patient comfort [[9], [10], [11]].

In this case series, we highlight the clinical versatility of HVNI by presenting three critically ill patients in whom this modality was deployed during the respiratory weaning phase to achieve a Individualized clinical objective in each case. Written informed consent was obtained from all patients (or their legal representatives) for the publication of clinical data in accordance with institutional ethical standards. All therapeutic decisions were made in adherence to current international guidelines on noninvasive respiratory support in the ICU [7,12].

## 2 Case presentations

### 2.1 Case 1: Prophylactic stabilization to prevent post-extubation failure

**Objective:** Safe transition from invasive mechanical ventilation to spontaneous breathing in a high-risk patient.

**Clinical course:** A 78-year-old male with a history of severe COPD was intubated following an episode of acute hypercapnic respiratory failure (pCO_2_ 68.8 mmHg) complicated by cardiac arrest. After successful initial management, the patient was extubated on day one. Given the well-documented high risk of post-extubation failure in severe COPD patients—with reintubation rates exceeding 20% in high-risk populations—he was immediately transitioned to prophylactic HVNI (flow 45 L/min, FiO_2_ 50–60%) [13].

**Outcome:** The primary objective of preventing post-extubation respiratory failure was achieved. The patient maintained a remarkably stable arterial pH (7.40–7.41) over the first 24 hours, alongside steady pCO_2_ levels and successful metabolic lactate clearance, without any signs of respiratory distress or need for escalation.

**Evidence context:** The prophylactic application of high-flow oxygen therapies immediately post-extubation is strongly supported by current evidence. A systematic review and meta-analysis demonstrated that HFNC significantly reduces reintubation compared with conventional oxygen therapy (RR 0.46; 95% CI 0.30–0.70) [4]. The American College of Chest Physicians/American Thoracic Society guidelines recommend noninvasive respiratory support to prevent post-extubation respiratory failure in high-risk patients [14]. Furthermore, the combination of high-flow nasal oxygen with NiPPV has been shown to further reduce reintubation rates in high-risk populations (11.8% vs. 18.2%) [15]. HVNI, through its efficient dead space washout and accurate FiO_2_ delivery, represents an ideal post-extubation bridge in this clinical scenario [9,10].

### 2.2 Case 2: Rescue therapy for interface intolerance and secretion clearance

**Objective:** Providing a comfortable alternative to NiPPV to allow active airway clearance while maintaining adequate gas exchange.

**Clinical course:** A 77-year-old male was intubated for acute respiratory arrest secondary to bilateral pulmonary embolism and severe mucous plugging. Upon extubation, he was placed on standard NiPPV. However, the patient developed persistent mild alkalosis (pH ≥ 7.54) and, critically, was unable to clear dense bronchial secretions due to the continuous positive pressure mask interface, which precluded the use of mechanical in-exsufflation devices.

**Outcome:** The clinical objective shifted toward patient comfort and active secretion management. The patient was transitioned to HVNI (45 L/min, FiO_2_ 70%). This open-interface system maintained gas exchange completely equivalent to NiPPV, but crucially allowed the simultaneous use of mechanical in-exsufflation ("Cough Assist") to actively clear the airways. The patient tolerated the transition without hemodynamic or respiratory compromise.

**Evidence context:** Multiple randomized clinical trials have established that HVNI is non-inferior to NiPPV in treating acute hypercapnic respiratory failure, with similar effects on pCO_2_ and pH over time [5,9,10]. Importantly, patients consistently report significantly better comfort levels with HVNI compared to NiPPV at multiple time points (p = 0.003), and nasal high-flow users demonstrate significantly lower levels of dyspnea and discomfort [10,11]. The open-interface design of HFNC systems facilitates mucociliary clearance through optimal humidification and allows uninterrupted access to the airway for adjunctive therapies—a critical advantage when traditional mask interfaces are detrimental to the patient's recovery [4,5,8].

### 2.3 Case 3: Bridging functional recovery in prolonged tracheostomy weaning

**Objective:** Enabling physical mobilization, natural upper airway conditioning, and functional rehabilitation in a complex tracheostomized patient.

**Clinical course:** A 55-year-old male suffered from severe necrotizing pancreatitis complicated by septic shock and acute kidney injury. Due to a prolonged and difficult weaning process, a percutaneous tracheostomy was performed. After multiple failed ventilator weaning trials, a rehabilitative approach was required.

**Outcome:** To rehabilitate the upper airways, the tracheostomy cannula was decuffed and capped, and respiratory support was shifted to the upper airways via nasal HVNI at 45 L/min and 50% FiO_2_. The patient immediately achieved adequate gas exchange, autonomic hemodynamic stability, and no signs of respiratory fatigue. This setup successfully allowed the patient to be physically mobilized (sitting at the edge of the bed) and permitted the resumption of oral feeding on the same day—milestones that would have been significantly hindered by continued invasive ventilation.

**Evidence context:** The REDECAP trial demonstrated that continuous high-flow oxygen therapy in tracheostomized patients significantly shortened the time to decannulation (median 6 vs. 13 days; absolute difference 7 days; 95% CI 5–9), with lower incidence of pneumonia and tracheobronchitis [1]. High-flow therapy via tracheostomy with a one-way speaking valve has been shown to be well tolerated and safe, with the added benefit of humidification enabling extended upper airway use without mucosal desiccation [15]. The open-interface nature of HFNC systems allows patients to eat, drink, and communicate during therapy, facilitating early mobilization and functional recovery that would otherwise be hindered by invasive ventilation setups [5,8]. In this case, the innovative application of nasal HVNI with a capped tracheostomy served as an effective rehabilitative bridge, conditioning the upper airways while maintaining respiratory support.

## 3 Discussion

While HVNI relies on a unified set of physiological mechanisms—high-velocity dead space washout, accurate FiO_2_ delivery, mild PEEP generation, and enhanced mucociliary clearance—this case series demonstrates that its clinical application is highly adaptable to diverse weaning scenarios [2,4,5]. The three cases presented illustrate how the same technology can be strategically deployed to achieve fundamentally different clinical objectives: preventing immediate post-extubation failure in a high-risk COPD patient, rescuing a patient from NiPPV intolerance to enable active secretion clearance, and facilitating physical rehabilitation in a complex tracheostomy weaning scenario.

Several features of HVNI make it particularly suited for this versatility. Its open-interface design preserves patient comfort and allows concurrent interventions such as airway clearance maneuvers, oral fluid intake, and physical mobilization [5,8]. Its non-inferiority to NiPPV in managing hypercapnic respiratory failure has been demonstrated across multiple randomized trials, providing a robust evidence base for its use as an alternative when NiPPV is not tolerated or is clinically suboptimal [5,[9], [10], [11]].

This case series has inherent limitations, including the small sample size, the absence of a control group, and the observational nature of the data. Nevertheless, these cases provide hypothesis-generating evidence supporting the role of HVNI as a versatile and efficacious tool in the intensivist's armamentarium for individualized respiratory weaning strategies.

## 4 Conclusion

HVNI provides a versatile, well-tolerated, and evidence-supported modality for respiratory weaning in critically ill patients. Whether the clinical objective is preventing post-extubation failure, rescuing a patient from NiPPV intolerance to clear secretions, or facilitating functional rehabilitation in a tracheostomized patient, HVNI can be strategically adapted to meet distinct clinical needs. Future prospective studies are warranted to further define the optimal role of HVNI across the spectrum of ICU weaning scenarios.

## CRediT authorship contribution statement

**Iacopo Cappellini:** Conceptualization, Investigation, Project administration, Writing – original draft. **Vittorio Pavoni:** Conceptualization, Formal analysis, Investigation, Writing – original draft.

## Ethical approval and consent to participate

Written informed consent was obtained from all patients (or their legal representatives) for publication of clinical data in accordance with institutional ethical standards.

## Consent for publication

Written informed consent for publication was obtained from all patients (or legal representatives).

## Availability of data and materials

Not applicable.

## Funding

None.

## Declaration of competing interest

The authors declare no competing interests.
